# Adult hippocampal neurogenesis reduces memory interference in humans: opposing effects of aerobic exercise and depression

**DOI:** 10.3389/fnins.2013.00066

**Published:** 2013-04-30

**Authors:** Nicolas Déry, Malcolm Pilgrim, Martin Gibala, Jenna Gillen, J. Martin Wojtowicz, Glenda MacQueen, Suzanna Becker

**Affiliations:** ^1^Department of Psychology, Neuroscience and Behaviour, McMaster UniversityHamilton, ON, Canada; ^2^Department of Kinesiology, McMaster UniversityHamilton, ON, Canada; ^3^Department of Physiology, University of TorontoToronto, ON, Canada; ^4^Department of Psychiatry, University of CalgaryCalgary, AB, Canada

**Keywords:** hippocampus, neurogenesis, exercise, depression, interference, pattern separation

## Abstract

Since the remarkable discovery of adult neurogenesis in the mammalian hippocampus, considerable effort has been devoted to unraveling the functional significance of these new neurons. Our group has proposed that a continual turnover of neurons in the DG could contribute to the development of event-unique memory traces that act to reduce interference between highly similar inputs. To test this theory, we implemented a recognition task containing some objects that were repeated across trials as well as some objects that were highly similar, but not identical, to ones previously observed. The similar objects, termed lures, overlap substantially with previously viewed stimuli, and thus, may require hippocampal neurogenesis in order to avoid catastrophic interference. Lifestyle factors such as aerobic exercise and stress have been shown to impact the local neurogenic microenvironment, leading to enhanced and reduced levels of DG neurogenesis, respectively. Accordingly, we hypothesized that healthy young adults who take part in a long-term aerobic exercise regime would demonstrate enhanced performance on the visual pattern separation task, specifically at correctly categorizing lures as “similar.” Indeed, those who experienced a proportionally large change in fitness demonstrated a significantly greater improvement in their ability to correctly identify lure stimuli as “similar.” Conversely, we expected that those who score high on depression scales, an indicator of chronic stress, would exhibit selective deficits at appropriately categorizing lures. As expected, those who scored high on the Beck Depression Inventory (BDI) were significantly worse than those with relatively lower BDI scores at correctly identifying lures as “similar,” while performance on novel and repeated stimuli was identical. Taken together, our results support the hypothesis that adult-born neurons in the DG contribute to the orthogonalization of incoming information.

## Introduction

The hippocampus, a structure in the medial temporal lobe, has long been implicated in the encoding and retrieval of contextual and associative memories. More specifically, the hippocampus is thought to be required for laying down distinctive memory traces for highly similar events, a process known as pattern separation. Marr's (1971) computational model attributed this orthogonalizing function specifically to the dentate gyrus (DG) sub-region of the hippocampus (Marr, 1971). The CA3, on the other hand, via its extensive recurrent collateral connections, was hypothesized to be ideally suited for binding incomplete or degraded components of an event into a coherent memory (pattern completion) (Marr, 1971). This pattern completion capability is important for memory retrieval, but may also lead to errors in recognition.

There are several unique electrophysiological and anatomical features of the DG that led Marr to hypothesize its crucial role in pattern separation. There are many more principle neurons in the DG than in layer II of the entorhinal cortex (EC), its major source of input (a 5 to 10-fold increase in the rat, for example, Amaral et al., 1990). Furthermore, DG granule cells have much lower activity levels and higher spatial selectivity than other hippocampal regions (Barnes et al., 1990; Jung and McNaughton, 1993). This highly divergent, ultra-sparse neural code in the DG is thought to be crucial for its ability to perform pattern separation. Unit recordings from DG cells lend further support to the hypothesized role of the DG in pattern separation, although the exact mechanism of this process at the cellular level remains unclear (Leutgeb et al., 2007).

Another unique property of the DG is that it undergoes neuronal turnover throughout the lifespan. This ongoing process of neurogenesis may contribute further to the proposed function of keeping similar memories distinct, thereby minimizing interference (Becker, 2005). There is mounting empirical support from rodent studies for the role of DG neurogenesis in minimizing interference between overlapping memories. For example, animals with reduced neurogenesis are impaired at distinguishing between highly similar spatial cues (Clelland et al., 2009), learning overlapping odor pair discriminations (Luu et al., 2012) and long-term retention of a learned visual discrimination when an interfering visual task is performed subsequently (Winocur et al., 2012). Conversely, exercise upregulates neurogenesis and has been shown to enhance performance on memory tasks that have a high interference component (Creer et al., 2010; Sahay et al., 2011; Winocur et al., 2012). Moreover, DG neurogenesis is not only critical for memory, but may play an important role in stress and mood regulation. For example, neurogenesis seems to be important for buffering the stress response (Snyder et al., 2011) and mediating the effects of antidepressants (e.g., Santarelli et al., 2008).

Given the critical role that DG neurogenesis seems to play in memory and mood regulation in rodents, it would be of tremendous interest to confirm the functional role of neurogenesis in the human brain. Unfortunately, there is currently no way to directly and non-invasively assay neurogenesis *in vivo*. A few studies have employed magnetic resonance imaging (MRI)-based methods to assay putative correlates of neurogenesis. For example, Manganas et al. (2007) used MR spectroscopy to measure a 1.28 ppm peak that they claim represents neural progenitor cells. Pereira et al. (2007) used contrast enhanced MRI to track DG blood volume changes induced by exercise. Importantly, 12 weeks of exercise lead to increased DG blood volume in both mice and humans, and in mice the DG (blood) volume increase was further shown to correlate with increased neurogenesis. Such MRI-based methods hold promise and merit further research. Another approach is to examine lifestyle factors known to correlate with neurogenesis. For example, aerobic exercise has been shown to upregulate neurogenesis (van Praag et al., 1999a,b, 2005; Olson et al., 2006; Pereira et al., 2007; Fabel et al., 2009), whereas high levels of stress, alcohol bingeing and BDNF polymorphisms are associated with reduced DG cell proliferation and/or survival (Cameron and Gould, 1994; Jang et al., 2002; Mirescu and Gould, 2006; Warner-Schmidt and Duman, 2006; Morris et al., 2010; Bath et al., 2012).

The approach we have taken is to examine performance on human analogues of tasks found to be neurogenesis-dependent in rodents, and assay lifestyle factors known to correlate with neurogenesis. We can thus test hypotheses concerning the functional role of neurogenesis in the human brain. As a first step in this direction, we previously examined the association between stress, depression and performance on several subscales of the Cambridge neuropsychological test automated battery (CANTAB®) battery. Based on the well-accepted role of stress in the pathogenesis of human depression (Brown et al., 1999), and the observation that chronically stressed animals have suppressed neurogenesis (Cameron and Gould, 1994; McEwen, 2001), it was predicted that individuals who have elevated scores on the Beck Depression Inventory (BDI) would have suppressed neurogenesis, and exhibit selective memory deficits on neurogenesis-dependent memory tasks. The CANTAB delayed match to sample (DMS) task at long delays was predicted to be sensitive to neurogenesis because it has many of the characteristics of neurogenesis-dependent tasks identified in rodent studies. It requires participants to encode complex configural visual patterns, and later discriminate between the studied patterns and highly similar lures. Rodents with reduced neurogenesis are impaired at visual recognition memory on DNMS task at long delays (Winocur et al., 2006), and when there is high visual similarity or spatial similarity between the targets and lures (Clelland et al., 2009; Creer et al., 2010); all of these tasks have a high interference component, and place high demands on the pattern separation capabilities attributed to the DG. We found that young adults with elevated BDI scores were selectively impaired on the DMS task at long delays, in spite of intact performance on DMS at shorter delays as well as on a large battery of other memory tests (Becker et al., 2009). Here, we sought to extend these findings to another test that may require adult neurogenesis, the visual pattern separation task developed by Kirwan and Stark (2007). This task has been used in several human fMRI studies (e.g., Kirwan and Stark, 2007; Bakker et al., 2008; Yassa et al., 2011) with results that are consistent with the notion of the DG being responsible for pattern separation. Specifically, the DG/CA3 subregion is strongly activated by both novel items and highly similar lures, but much less active for repeated items (Kirwan and Stark, 2007).

In the first experiment, we tracked performance on the visual pattern separation task as a function of a 6-week exercise training intervention in previously sedentary but otherwise healthy young adults. As noted above, exercise causes chronic elevations in DG blood volume in both mice and humans (Pereira et al., 2007) and is a well-established up-regulator of neurogenesis in rodents (van Praag et al., 1999a,b, 2005; Olson et al., 2006; Fabel et al., 2009). In the second experiment, we examined the relationship between performance on this task and depression scores. We predicted that a participant's ability to identify “similar lures,” that is, images of objects that are highly similar to previously studied ones, would require hippocampal neurogenesis for optimal performance. Therefore, pattern separation performance was predicted to be enhanced in exercisers, particularly in those who exhibited large changes in fitness, and impaired in those with elevated depression scores.

## Experiment 1

### Methods

#### Participants

All aspects of our aerobic exercise training study were approved by the Hamilton Integrated Research Ethics Board (HIREB). We recruited 13 healthy but sedentary young adult participants from the McMaster University student population using ethics board approved advertisements that were posted across campus. All participants provided written informed consent and met the inclusion criteria for our study: a healthy body mass index (≤25), the requirements for beginning physical activity: a sedentary lifestyle (no more than 1 h of physical activity per week) and no prior history of psychiatric illness. Two of the 13 participants did not take part in the high intensity interval training (HIT) aspect of the program, but participated in all other aspects of the study, pre- and post-training. A week prior to the commencement, and a week following the completion of a 6 week HIT program, an intervention previously shown to be effective for improving aerobic fitness (Hickson et al., 1977), each participant performed a battery of cognitive tests and a VO_2peak_ test of fitness.

#### Cognitive testing

***Putative neurogenesis-dependent task.*** A cognitive task that generates high levels of interference between previously learned and subsequently tested stimuli is Kirwan and Stark's (2007) “pattern separation task,” a three alternative forced choice visual recognition memory task that includes some objects that are repeated across trials, which are termed “repetitions,” some images that are highly similar, yet not identical to ones previously viewed that are called “lures” and some completely novel objects, which are termed “foils.” The version of the task that we used here was adapted from Kirwan and Stark's (2007) task, whereby participants are shown a series of 88 images of familiar everyday objects (32 first presentations, 16 old “repetitions,” 16 similar “lures,” and 24 unrelated “foils”). Images are presented one at a time in pseudorandom order for 2500 ms each with a 500 ms inter-trial interval, with the constraint that there is an average distance of 30 items separating any first presentation from either a subsequent repetition or from a subsequent similar object. Following a brief delay, a second series of 112 images (48 repetitions, 48 lures, 16 foils) is presented and the participant is asked to judge whether each object is “old,” “similar” to an object presented in the previous set, or “new.” Correct responses are made when repetitions are identified as “old,” when lures are correctly classified as being “similar” but not identical to the target stimulus, and when unrelated foils are classified as “new.” Thus, the correct identification of a lure stimulus constitutes a correct rejection, whereas incorrectly classifying a lure stimulus as “old” constitutes a false positive (i.e., falsely identifying a lure stimulus as being identical to the target stimulus). The presentation and recognition phases are then repeated in a second block of trials, using an entirely different set of stimuli. As mentioned, Kirwan and Stark's object recognition task involves discriminating between previously learned patterns (targets) and highly similar lures, a potential source of interference, and may thus benefit from the so-called “pattern separation” capability attributed to the DG (Marr, 1971; McClelland et al., 1995; Kesner, 2007), and more specifically to neurogenesis (Becker, 2005; Aimone et al., 2006; Appleby and Wiskott, 2009; Becker et al., 2009). We therefore posit that participants with putatively higher rates of neurogenesis in the DG will be able to better overcome interference and, consequently, outperform those with relatively lower levels of neurogenesis at correctly identifying lure stimuli as “similar.” On the other hand, we do not expect to see any measurable difference in performance between groups on conditions that do not likely require adult-born neurons in the DG. Specifically, both high and low neurogenesis groups should perform the same when it comes to classifying exact repetitions of previously viewed object as “old” or when categorizing novel stimuli as “new.” If one possesses a lower level of newborn cells in the DG than is required for optimal task performance, we would expect that the CA3, a region of the hippocampus thought to be important for binding information, will dominate processing and lead to an increased number of pattern completion errors. In terms of the pattern separation task, the misidentification of a lure stimulus as “old”—a false positive—would constitute a pattern completion error.

***Putative neurogenesis-independent task.*** As a control task, we sought a memory test that is known to be hippocampal dependent but was not predicted to be neurogenesis-dependent. We chose the paired associate learning (PAL) task from CANTAB®, a visuo-spatial associative learning task which is well-established to be sensitive to hippocampal pathology, but lacks a high-interference component that may rely heavily on DG neurogenesis. We previously showed that young adults with elevated depression scores performed identically to non-depressed participants on the PAL task (Becker et al., 2009). In this task, in each trial, 6 white squares are displayed on the computer screen. One at a time, each square momentarily disappears to reveal what is hiding underneath—either nothing or an abstract object. Once the content of each square is known, the participant is presented with another series of abstract objects, one at a time, and now must appropriately select the white square that is occluding the target object being shown. With each series, the task becomes progressively more difficult as the number of abstract objects hidden underneath the squares increases from 1 to 6. In the final iteration of the task two more white squares are added and the participant must try to remember the location of 8 unique objects.

#### Aerobic exercise

**VO_2peak_*testing***. Peak oxygen uptake (VO_2peak_) is the gold-standard measurement of aerobic fitness and is obtained by measuring oxygen uptake continuously during an incremental test to exhaustion. A baseline VO_2peak_ score was obtained from each participant during the week prior to the start of exercise. A second VO_2peak_ score was then obtained from each participant in the week following our chronic exercise intervention (described below).

***High-intensity interval training intervention.*** Gormley and colleagues (2008) found that with the volume of exercise controlled, it was those who exercised at near-maximal intensity that achieved the greatest increase in aerobic VO_2peak_. Volume of exercise was controlled by multiplying the intensity (% of heart rate reserve or % of VO_2_ reserve) of the exercise program by the duration, in minutes, and the frequency, in days (Gormley et al., 2008). Accordingly, we used a 6 week HIT program comparable to the near-maximal intensity condition used by Gormley et al. (2008) except that our exercisers ran on an outdoor track rather than using stationary cycles. In each week of the program participants ran in three sessions that varied in both duration and intensity as outlined below. Before and after each session, subjects completed 5 min of running at 50% of heart rate reserve (HRR) as well as sport-specific stretching in order to warm-up and -down. Each session was also separated from the next one by at least 24 h in order to promote recovery. In week 1, participants ran for 15 min sessions at 65% of HRR, while in week 2 they ran in 20 min sessions at 65% of HRR. In weeks 3–6 we had runners start at 75% of HRR for 5 min, followed by alternating sprint and rest phases of 95% HRR and 50% HRR, respectively. The number of sprints in each session started at 3 on week 3, and increased by 1 in each subsequent week. Participant heart rate information was displayed on Timex Ironman® Road Trainer™ watches and was monitored periodically throughout each training session by one of the experimenters.

#### Statistical analyses

For all correlative analyses Pearson's *r* was used. The participants in our exercise study were split into two groups based on their median change in VO_2peak_. Accordingly, those who experienced a change in VO_2peak_ below the median were considered low responders to exercise, while those with a change in VO_2peak_ greater than the median were considered high responders. Low and high responders to exercise were compared using the non-parametric Mann–Whitney *U*-test (which does not require the assumption of equal variances, because there were two non-exercisers in the former group), while paired samples *t*-tests were used for within group repeated measures comparisons. Results are presented as mean (*M*) ± standard deviation (*SD*). For each statistical test a *p* value (two-tailed) ≤ 0.05 was considered significant, except where otherwise stated.

### Results

#### Aerobic fitness and pattern separation performance

Correctly identifying unrelated foils as “new” in our visual pattern recognition task is relatively easy, with participants typically scoring near ceiling (*M* = 89%, *SD* = 11.1%). We therefore considered any participant who scored more than two standard deviations away from the mean on these items to have not properly understood or disregarded the task instructions, or to have implemented a deviant response strategy (e.g., answering only “old” or “similar” for every object displayed in the recognition phase, completely ignoring the “new” response option). On this basis, 1 participant's data was discarded, leaving 12 participants' data that were included in subsequent analyses (4 male, 8 female; mean age = 21.83, *SD* = 2.25). Six weeks of highly controlled physical activity had a highly significant impact on fitness, as revealed by a paired samples *t*-test of VO_2peak_ pre- vs. post-exercise [*t*_(9)_ = 4.10, *p* = 0.003]. However, a high degree of variability in changes to VO_2peak_ following chronic exercise is common among previously sedentary participants (Hickson et al., 1977). Indeed, individuals in our study varied widely in their response to the fitness intervention, ranging from no change to a 29% improvement in fitness. Therefore, we split the group into exercise responders (*N* = 6, *M* = 14%, range = 9–29%) and non-responders (*N* = 6, *M* = 3%, range = −3 to 8%) based on the median change in VO_2peak_, and then analyzed differences in cognitive task performance between these two groups. Accordingly, those who experienced a change in VO_2peak_ of 8% or less were considered low responders to exercise, while those with a change of 9% or greater were considered high responders. We included in the non-responder group two individuals who did not complete the exercise program, but completed all pre- and post-six week cognitive and fitness testing. Prior to making any comparisons between the change in fitness and the change in performance on the visual object recognition task pre- vs. post-exercise, we applied a correction (as described in Yassa et al., 2011) in order to control for response bias across groups. For example, the (uncorrected) probability of judging an old item as “old” could be inflated if one judged every stimulus as “old.” Therefore, we subtracted the proportion of “old” responses given the presentation of an unrelated foil (new item) from the proportion of correct “old” responses given a repetition (old item):

Formula 1. [p(“Old”|Target)−p(“Old”| Foil)]

Similarly, we subtracted the proportion of “similar” responses given the presentation of a foil (new item) from the proportion of correct “similar” responses given the presentation of a lure (similar item):

Formula 2. [p(“Similar”|Lure)−p(“Similar”|Foil)]

**Figure 1:**
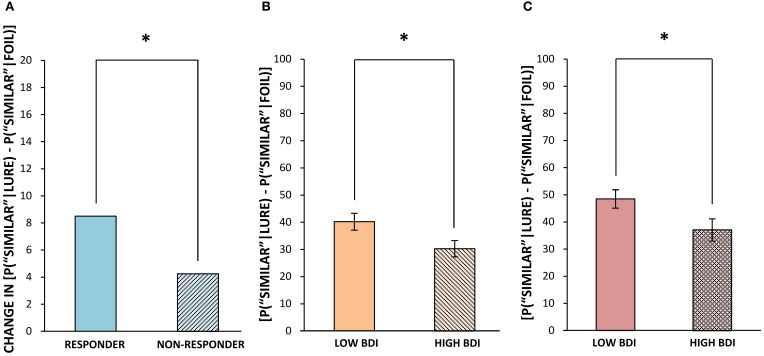
**Correct classification of similar lures pre- vs. post-exercise in Experiment 1 (A), and in those with below median and above median BDI scores in Experiment 2 (B,C). (A)** Median change in % correct identification of lures as “similar” (bias corrected ranked difference scores) following exercise for responders vs. non-responders. **(B)** Mean % correct identification of lures as “similar” (bias corrected, ± standard error) when the original target appeared in the same block. **(C)** Mean % correct identification of lures as “similar” (bias corrected, ± standard error) when the target appeared in a previous block. ^*^p ≤ 0.05.

The application of Formulae 1 and 2 resulted in the correction for a participant's bias to select “old” or “similar,” respectively. Thus, it was these bias-corrected scores that were used for the following analysis. As previously mentioned, it was expected that exercise would cause increased levels of hippocampal neurogenesis, leading to an enhanced ability to correctly classify lure objects as “similar.” Indeed, when comparing the average difference scores of responders and non-responders on the pattern separation task post- minus pre-exercise, we found that it was specifically those with a higher change in fitness (and putatively a larger increase in hippocampal neurogenesis) who demonstrated an improved ability to correctly identify lure stimuli as “similar,” going from 60% (*SD* = 18.77) correct before exercise to 71% (*SD* = 18.73) correct following exercise [*t*_(5)_ = 2.62, *p* = 0.05]. The exercise responders' improved accuracy at appropriately classifying lures as “similar” (evidence of pattern separation) was mirrored by a concurrent reduction in the number of misidentifications of lures as “old” (evidence of pattern completion) post- vs. pre-exercise [*t*_(5)_ = 3.16, *p* = 0.03]. On the other hand, non-responders to exercise began by correctly identifying lures as “similar” 69% (*SD* = 13.76) of the time and post-exercise remained at 69% (*SD* = 12.63) accuracy. Thus, there was a significant difference between exercise responders and non-responders in their post-training improvement at correctly classifying lures as “similar,” with a change of 11% (*SD* = 10.26) in high responders and 0% (*SD* = 5.19) in non-responders (*z* = 2.08, *p* = 0.04, Figure 1A). Since non-responders were slightly more accurate than responders at baseline (not significant), it could be that they also had inherently higher levels of fitness and neurogenesis at the outset. Indeed, although not significant, the non-responders were marginally more fit than responders at the outset of training, as their mean VO_2peak_ score was 114% (*SD* = 9.00) of their expected values, while responders' mean VO_2peak_ score was 105% (*SD* = 15.63) of their expected values prior to commencing our 6 week exercise regime [*t*_(10)_ = 1.29, *p* = 0.23]. This variability in baseline fitness could explain why non-responders failed to show any post-exercise improvement in either VO_2peak_ or in pattern separation performance.

Yassa and colleagues (2011) have reported that aging, which is associated with lower rates of hippocampal neurogenesis in many species (e.g., Klempin and Kempermann, 2007; Johnson et al., 2010; Knoth et al., 2010), is accompanied by a shift in the pattern separation vs. pattern completion tuning curve in CA3, such that larger changes in input (greater dissimilarity) are required for pattern separation to occur. Since lures may vary in their degree of similarity to the original targets, those that are most similar to the original target stimulus should generate maximal interference and thus present a higher opportunity for pattern completion to dominate processing and lead to additional false positives. If this shift in pattern separation vs. pattern completion behavior is dependent on the rate of adult hippocampal neurogenesis, then it would stand to reason that responders and non-responders to chronic exercise should also vary in their relative change in position on the tuning curve following exercise, such that responders demonstrate an increased bias toward pattern separation, whereas non-responders demonstrate a bias toward pattern completion. Furthermore, their ability to orthogonalize overlapping stimulus inputs should also depend on the degree of similarity between targets and lures. Therefore, we had a separate cohort of participants rate the similarity of each pair of target and lure stimuli from our pattern separation task on a 5 point likert scale, with 5 being most similar and 1 being least similar. Taking a median split of their average similarity ratings for each pair of target and lure objects allowed us to classify each set as “more similar” or “less similar.” In order to examine if the discrepancy in pattern separation performance between the exercise responders and non-responders was dependent on the degree of similarity between the target stimulus and the lure, we performed separate *post-hoc* analyses of performance on these two categories of items (“more similar” or “less similar”). For less similar lures, exercise responders showed a significant 12% (*SD* = 9.58) improvement in performance [*t*_(5)_ = 2.94, *p* = 0.02 (one-tailed)] at appropriately classifying “less similar” items as “similar” following exercise, going from 62% (*SD* = 15.93) to 74% (*SD* = 22.73) correct, while non-responders did not experience any change, remaining at 71% correct. Exercise responders showed a trend toward improved performance at correctly identifying “more similar” items as “similar” post-exercise a 10% (*SD* = 13.98) improvement, [*t*_(5)_ = 1.77, *p* = 0.07 (one-tailed)] going from 58% (*SD* = 18.56) to 68% (*SD* = 15.60) correct, which was mirrored by a significant decrease in the number of misidentifications of “more similar” lures as “old” [*t*_(5)_ = 3.80, *p* = 0.01 (one-tailed)]. On the other hand, the non-responder group experienced a mere 1% (*SD* = 8.82) change in the negative direction [*t*_(5)_ = 0.33 *p* = 0.38 (one-tailed)] by going from 67% (*SD* = 13.97) to 66% (*SD* = 11.48) correct. Overall, our data suggest that: (1) 6 weeks of aerobic exercise is sufficient to normalize and/or improve pattern separation performance in those who experience a relatively large change in fitness, perhaps by increasing the availability of young neurons in the DG that, in turn, act to reduce interference between highly similar objects; (2) the exercise-dependent increase in pattern separation performance is coupled with fewer pattern completion errors (misidentifying similar items as “old”); and finally (3) the superior ability to correctly reject a lure stimulus as “similar” following exercise holds true regardless of how similar the two stimuli are. Importantly, there were no significant changes in performance within or between high and low responder groups at correctly identifying repetitions as “old,” or in correctly identifying foil objects as “new.” There was also no significant difference between groups in their performance on the CANTAB® PAL task. Therefore, the improved ability of exercise responders to correctly recognize a lure item as being similar to, but not the same as, the target stimulus is likely due to a change in their ability to overcome interference, rather than a generalized improvement in memory functions.

#### Correlations between fitness and neurogenesis-dependent memory

**Figure 2:**
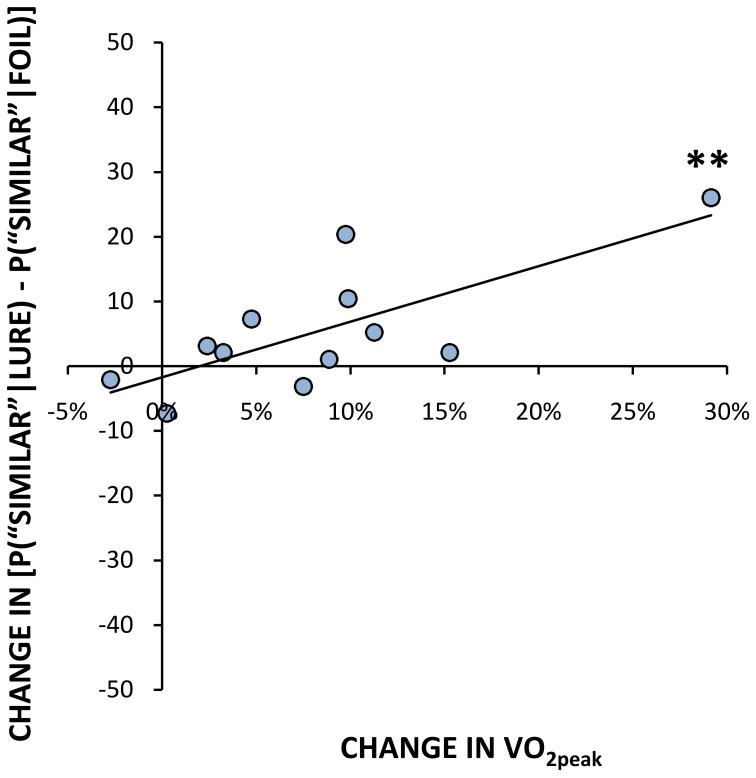
**Percent change in fitness scores (VO_2peak_) vs. change in mean % correct identification of lures as “similar” following exercise (*r* = 0.74, *p* < 0.01).** ^**^p ≤ 0.01.

In addition to analyzing between-group differences in participants who responded most vs. least to the exercise program, we examined their change in fitness levels (VO_2peak_ post- minus pre-exercise) as a continuous function of pattern separation performance. The change in fitness was positively correlated with the percent change in correctly identifying lures as “similar” [*r*_(10)_ = 0.74, *p* < 0.01, Figure 2]. In contrast, the change in VO_2peak_ negatively correlated with the percent change in “old” false positive responses to lure trials [*r*_(10)_ = −0.79, *p* < 0.01]. There was no correlation between change in VO_2peak_ and the percent change in miscategorizing lure objects as “new” following 6 weeks of physical activity. In other words, the more responsive that someone was to long-term aerobic training, represented by a relatively large change in fitness, the greater their improvement in response accuracy was on lure trials (i.e., correctly calling a similar object “similar”), while concurrently performing fewer pattern completion errors (i.e., incorrectly calling a lure stimulus “old”). Consistent with the comparisons between low and high responder groups, there was no significant correlation between change in VO_2peak_ and the change in ability to properly identify an unrelated foil as “new,” or between the change in VO_2peak_ and the change in ability to correctly identify a repetition as “old.” There was also no correlation between the change in VO_2peak_ following chronic exercise and the change in performance on PAL.

## Experiment 2

### Methods

#### Participants

The study protocol for our investigation of depression scores vs. learning and memory performance on our battery of cognitive tasks was approved by the HIREB. We recruited 57 healthy young adults from the McMaster University student population using an online experiment sign-up tool. All participants gave informed consent and met the inclusion criterion for our study, having no history or previous diagnosis of any psychiatric disorder. Participant data was made anonymous to the experimenters; however, a code linking their personal information to their BDI scores was given to a third party for assessment. If any student was flagged to be at risk for suicide or major depression based on their responses on the BDI, their contact information was forwarded to a psychological counselor at the McMaster Student Wellness Centre who would then contact them for voluntary counseling or to discuss alternative treatment options.

#### Cognitive and neuropsychological testing

**Figure 3:**
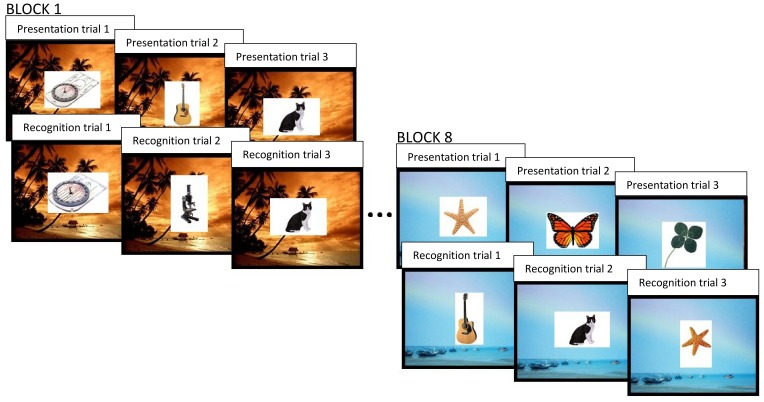
**The behavioral pattern separation task used in Experiment 2.** In each block, everyday objects are displayed 1 at a time in a sequence of presentation trials followed by a sequence of recognition trials. In recognition trials, the object can either be old (an exact repetition of a previously presented object), similar, but not identical, to a previous target, or a new, completely unrelated foil. Each block has a distinct visual context (background image). In recognition trials, for repetitions and similar items, the associated target may have been seen within the same block (same temporal and visual context) or within a previous block (different temporal and visual context).

Each participant first completed the BDI in a quiet, private testing room. Next, they completed a putatively neurogenesis-dependent task, which was a modified version of the pattern separation task used in Experiment 1, and the CANTAB® PAL task described above. In this experiment, we augmented the visual object pattern separation task used in Experiment 1 in order to assess the effects of learning across longer time delays and contextual changes. Specifically, we used 8 blocks of presentation and recognition phases, as opposed to the 2 blocks used in our chronic exercise study. Following each presentation and recognition phase (i.e., each block), there was a change in visual context, whereby the background display changed from one outdoor environment to another (see Figure 3). In each presentation phase, there were only 16 images of everyday objects and there were no repetitions or lures. The 2500 ms display time and 500 ms inter-trial interval remained unchanged, however, we introduced a white visual noise mask after the presentation of each stimulus. Each recognition phase had a different number of images being presented, depending on the block number. The first recognition phase included 8 repetitions of target stimuli, 8 similar lures and 22 unrelated foils. The recognition phases occurring in blocks 2 through 8 contained the same number of within block repetitions and lures; however, they also contained 1 repetition and 1 lure whose targets were originally presented in the previous block(s), as well as 2 additional novel objects. Therefore, as an example, the recognition phase in block 2 contained 8 repetitions and 8 lures whose target stimuli were originally shown in the immediately preceding presentation phase, just as in block 1; however, the second recognition phase also contained 1 repetition and 1 lure whose target images were first seen in the presentation phase of block 1, as well as 24 novel objects, as opposed to 22. In contrast, the recognition phase within the eighth block contained 8 repetitions and 8 lures of target stimuli that were shown in the eighth presentation phase, as well as 7 repetitions and 7 lures whose target stimuli were originally presented across each of the 7 preceding blocks, and 36 unrelated foils. Constructing the task in this way allowed us to vary the amount of interference between target stimuli and lures. Each target object was only seen once during a presentation phase, but an identical or similar item could appear either within the same block and in the same context (as in Experiment 1), or a varying number of blocks later, within a different context. This afforded us the opportunity to analyze differences in performance as a function of depression score and as a function of the degree of spatio-temporal context change between the presentation of the item and its preceding target.

#### Statistical analyses

For all correlative analyses Pearson's *r* was used. For all comparisons between participants scoring in the lower and upper range on the BDI questionnaire, we used the student's *t*-test, with equal variance assumed. For each statistical test a *p* value (two-tailed) ≤ 0.05 was considered significant, unless otherwise stated.

### Results

#### Depression and putative neurogenesis-specific cognition

**Figure 4:**
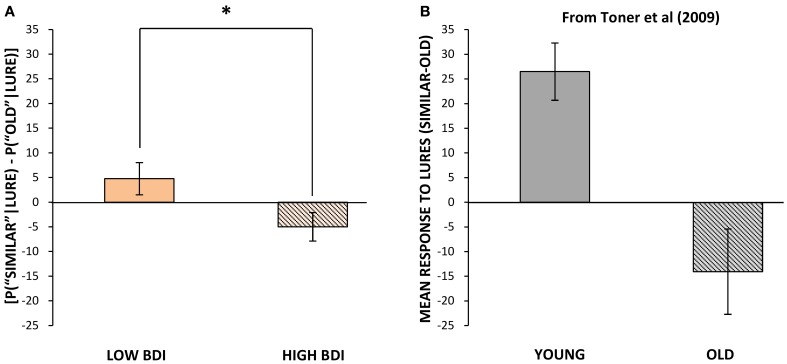
**Mean bias toward pattern separation vs. pattern completion (mean probability of correctly identifying lures as “similar” minus probability of misidentifying lures as “old,” ± standard error) vs. BDI score in Experiment 2 (A) and vs. age in Toner et al. (B). (A)** Mean bias scores when related target appeared in the same block as lure, for those with below median vs. above median BDI scores. **(B)** Mean bias scores for younger vs. older adults. Adapted from Figure 2 in Toner et al. (2009) and reproduced with permission from Cold Spring Harbor Laboratory Press. ^*^p ≤ 0.05.

***Performance within blocks.*** Performance on repetitions and lures that appeared within the same block and hence the same visual context as the original target (as was the case for all targets in Experiment 1) was analyzed separately, for ease of comparison with the results of Experiment 1. The same outlier screening method used in “Experiment 1” was applied here, resulting in the exclusion of 5 participants' data, leaving 52 participants who met criteria for inclusion in the analysis (13 male, 39 female; mean age = 19.48, *SD* = 2.05). In terms of self-reported levels of depression on the BDI in these participants, none scored in the severely depressed range (>29) and only 8 of 52 scored in the moderately depressed range (19–29). Therefore, we performed a median split based on the BDI scores to create two groups, low BDI scorers (BDI below 9; *N* = 27, mean BDI = 4.04, range = 0–8) and high BDI scorers (BDI 9 or greater; *N* = 25, mean BDI = 15.68, range = 9–29). These groups differed significantly in BDI score [*t*_(50)_ = 9.71, *p* < 0.001], but not significantly in age or gender. Next, we analyzed differences in cognitive task performance between individuals with lower vs. higher BDI scores. The same response bias corrections used in our first experiment were applied (see Formulae 1 and 2) prior to any further analyses. The low BDI group was superior to the high BDI group at correctly identifying lure items as “similar” in the visual pattern separation task, with mean scores of 40% (*SD* = 16.53) correct vs. 30% (*SD* = 15.45) correct, respectively [*t*_(50)_ = 2.23, *p* = 0.03, Figure 1B]. In order to compare our results to Toner and colleagues' (2009) findings in young vs. older adults, we calculated a measure of participants' bias toward pattern separation vs. pattern completion by subtracting the proportion of incorrect “old” responses to lure stimuli from the mean number of correct “similar” responses to lure trials. A more positive score would indicate that the participant made a higher proportion of correct “similar” responses to lure trials and thus pattern separation had dominated processing. On the other hand, a more negative score would indicate that more false positives, or incorrect, responses to lure trials had occurred and thus pattern completion had dominated processing. The low BDI group's scores were more biased toward pattern separation (as opposed to pattern completion) given the presentation of a lure stimulus, and were significantly higher than those of the high BDI group [*t*_(50)_ = 2.18, *p* = 0.03, Figure 4]. Importantly, there were no differences between the low and high BDI groups in their ability to recognize novel objects or repetitions.

As in Experiment 1, the similar lures were then divided, based on their subjective similarity ratings, into “more similar” and “less similar” lures. In doing so, it became apparent that the source of the low BDI group's superior performance on lure trials was primarily driven by their performance at correctly identifying “less similar” lures. Specifically, those with lower BDI scores correctly selected 45% (*SD* = 17.28) of “less similar” lures as “similar,” while those with higher BDI scores only identified 31% [*SD* = 18.50; *t*_(50)_ = 2.85, *p* < 0.01 (one-tailed)]. Those with lower BDI scores also made correspondingly fewer errors in calling “less similar” objects “old” as compared to those with higher BDI scores [*t*_(50)_ = 2.34, *p* = 0.01 (one-tailed)], whereas there was no difference between groups in the number of errors they made in miscategorizing “less similar” items as “new.” These data suggest that the low BDI group's superiority in correctly identifying the “less similar” lures as “similar” within blocks was rooted in fewer pattern completion errors. In contrast to the “less similar” items, the low BDI group only held a slight, non-significant advantage over those with high BDI scores at correctly identifying “more similar” lures as “similar” [*t*_(50)_ = 1.26, *p* = 0.11, (one-tailed)]. These results can be explained, in part, by the fact that both low and high BDI scorers performed relatively poorly at identifying the highly similar lures. Thus, the low BDI group performed better on “less similar” lure trials (45%, *SD* = 17.28) than they did on “more similar” lure trials [36%, *SD* = 16.84, *t*_(26)_ = 5.38, *P* < 0.001]. On the other hand, the high BDI group performed equally poorly at correctly identifying both “less similar” lures (30%, *SD* = 18.50) and “more similar” lures as “similar” [30%, *SD* = 15.80, *t*_(24)_ = 0.12, *p* = 0.90]. With “more similar” objects generating inherently higher levels of interference, these particularly difficult items may have been beyond the threshold for correct pattern separation, even in the low BDI group. On the other hand, those with high BDI scores (and putatively less neurogenesis) demonstrate a comparable deficit at identifying lures regardless of their similarity rating.

**Figure 5:**
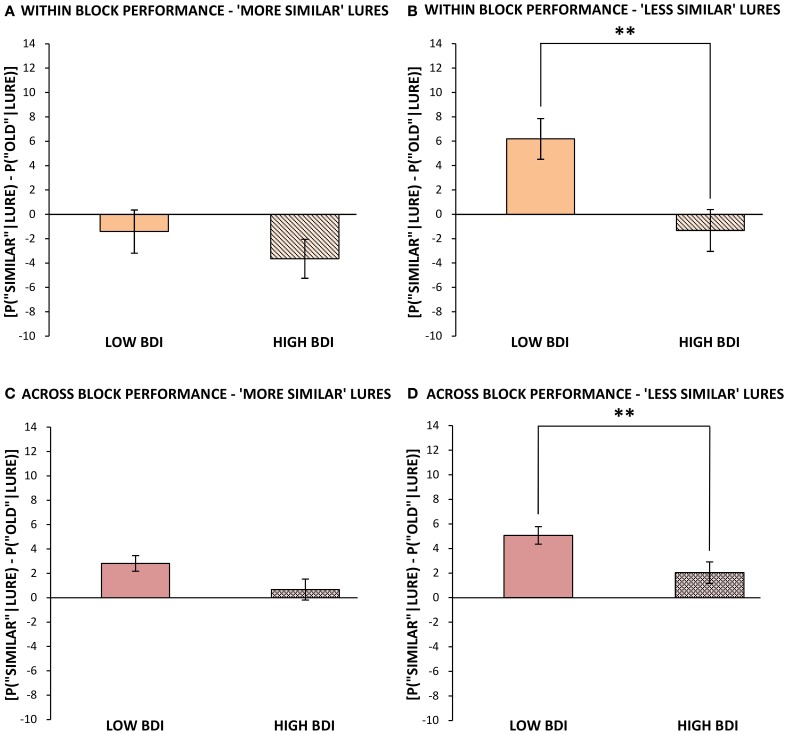
**Mean bias toward pattern separation vs. pattern completion (mean probability of correctly identifying lures as “similar” minus probability of misidentifying lures as “old,” ± standard error) in Experiment 2 for those with below median vs. above median BDI scores, (A) for “more similar” lures, when target was within the same block, (B) for “less similar” lures, when target was within the same block, (C) for “more similar” lures, when target appeared in a different block, and (D) for “less similar” lures, when target appeared in a different block.** ^**^p ≤ 0.01.

***Performance across blocks.*** The version of our visual pattern separation task used in Experiment 2 also contained some repetitions and lures that appeared across different blocks from the original presentation. Interestingly, both low and high BDI groups benefited from the increased temporal spacing between these items, and made significantly fewer errors in calling a lure stimulus “old” when it appeared in a different block, as opposed to the same block, from the target presentation. This effect did not depend on the similarity rating of the lure, as both “less similar” lures [Low BDI = −12%, *SD* = 15.35, *t*_(26)_ = 4.01, *p* < 0.001; High BDI = −12%, *SD* = 14.42, *t*_(24)_ = 4.31, *p* < 0.001] and “more similar” lures [Low BDI = −15%, *SD* = 16.16, *t*_(26)_ = 4.71, *p* < 0.001; High BDI = −12%, *SD* = 16.89, *t*_(24)_ = 3.47, *p* < 0.01] were less often misclassified as “old” (a repeat) when they were presented across blocks, as shown in Figures 5A–D. Thus, contextual changes occurring between the presentation and recognition trials improved the ability of both low and high BDI groups (although the difference was not significant in the higher BDI group) to correctly select lure images as “similar” [Low BDI = 8% increase, *SD* = 19.08, *t*_(26)_ = 2.25, *p* = 0.03; High BDI = 7% increase, *SD* = 17.96, *t*_(24)_ = 1.91, *p* = 0.07]. Evidently, the low BDI group had a resulting 11% advantage over the high BDI group at correctly calling a lure “similar” across blocks [*t*_(50)_ = 2.08, *p* = 0.04, Figure 1C]. Just as was the case within blocks, the high BDI group made significantly more pattern completion errors on lure trials than did the low BDI group, or conversely, the low BDI group displayed superior pattern separation [*t*_(50)_ = 2.87, *p* = 0.01].

As before, trials on which the lure appeared across blocks from the original target presentation were subdivided into two categories based on each lure's similarity rating into “less similar” and “more similar.” There was no difference between the low and high BDI groups in the number of errors they made in calling a similar object “new,” regardless of how similar the object was. In contrast, and comparable to the relationship described within blocks, those with lower BDI scores outperformed those with higher BDI scores at identifying “less similar” lures that appeared across blocks [*t*_(50)_ = 2.14 *p* = 0.02 (one-tailed)]. Interestingly, those with low BDI scores also exhibited superior performance at correctly identifying the “more similar” lures as “similar” when they appeared in a later block [*t*_(50)_ = 1.82, *p* = 0.04 (one-tailed)]. The greater ability of low BDI scorers to accurately categorize the “less similar” lures within a different block from its original target presentation was mirrored by significantly fewer pattern completion errors, or false positives, compared to higher BDI scorers [*t*_(50)_ = 1.76, *p* = 0.04 (one-tailed)]. In contrast, the low BDI group's improved ability to correctly distinguish “more similar” lures from the original targets, when presented in a contextually distinct recognition phase, was not solely the result of a reduction in pattern completion errors; they showed a slight reduction in the number of errors in calling the “more similar” lures “old” as well as fewer errors in calling the “more similar” lures “new.”

Taken together, the data within and across blocks suggest that when targets and lures appear within the same visual and temporal context (Figures 5A,B), if pattern overlap is too high (Figure 5A), neurogenesis levels are not sufficiently high in either group to successfully separate the patterns. On the other hand, provided that targets and lures do not overlap too strongly (Figure 5B), depression levels (putatively reflecting neurogenesis levels) cause a shift in performance from successful pattern separation to erroneous pattern completion. In contrast, when there is a longer temporal spacing between similar items, coupled with a change in visual context (Figures 5C,D), participants' performance shifts more toward pattern separation, regardless of BDI score or target-lure similarity. One possible explanation for this finding is that the change in time and context led to forgetting of the original image, thereby promoting a general bias toward selecting “similar” or “new” as opposed to “old” when the lure appeared. Another possibility is that the contextual changes occurring between the presentation and recognition trials led to reduced interference between the original image and the lure by adding sufficient contrast to the lure stimulus for it not to be encoded as an “old” image. If the former explanation was true, then we might expect that incomplete or low resolution memories for the original stimulus would lead to more errors in calling a similar object “new,” but this was not the case. On the contrary, we found that the number of “new” misidentifications of lures when the target occurred in a different block was not significantly different in either group from their performance when the target occurred within blocks. Further, if there was a greater bias to select the “similar” response option when lures are presented in a different block from the original image, then there would be no significant effect after bias correction. Again, this was not the case. The second scenario, therefore, seems to be more strongly supported. We would expect that if time and contextual changes provide additional separation between the target stimulus and subsequent lure object, then performance should improve where pattern separation processes are needed. Indeed, we found that contextual changes occurring between the presentation and recognition trials improved the pattern separation abilities of both low and high BDI groups.

In sum, these data suggest that both within and across blocks: (1) those with lower BDI scores demonstrate superior performance at recognizing a lure as “similar,” especially when there is less overlap in salient features (i.e., when the objects are “less similar” to one another); (2) the low BDI group's advantage in distinguishing between “old” and “similar” items reflects enhanced pattern separation processes and fewer pattern completion errors; and (3) a longer amount of time in combination with a contextual shift between encoding and recognition may benefit not only those with putatively greater neurogenesis, but also those with a deficit in pattern separation and putatively less adult-born cells available in the DG (i.e., high BDI scorers) by adding contrast between stimuli, consequently aiding them to correctly identify a lure as “similar.” There was no difference between groups on the CANTAB® PAL task, further suggesting that the lower BDI group's superior performance on the pattern separation task is not due to a generalized advantage in memory.

#### Correlational analysis of depression and neurogenesis-dependent memory

**Figure 6:**
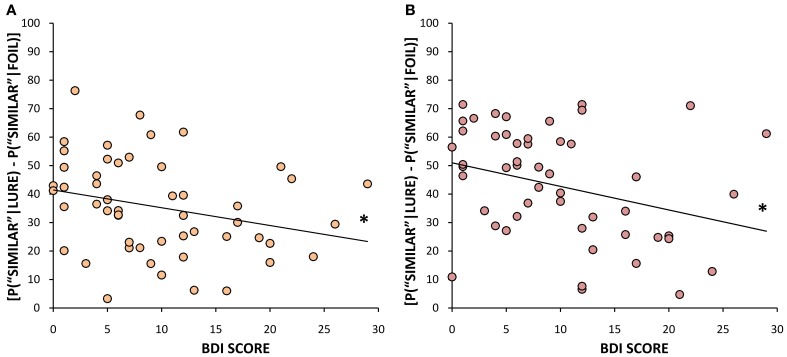
**Mean % correct identification of similar lures (bias corrected) as a function of BDI scores, (A) when they appear in the same block as the original target (*r* = −0.272, *p* = 0.05), (B) when they are presented in a different block from the original target (*r* = −0.297, *p* = 0.03).** ^*^p ≤ 0.05.

In addition to the analyses described above based on a median split of BDI scores, we also considered BDI scores as a continuous measure of depression levels, and performed correlational analyses between BDI scores and our memory measures. BDI scores negatively correlated with the proportion of correct identifications of lure objects as “similar” when they were presented within the same block as the original target [*r*_(50)_ = −0.272, *p* = 0.05, Figure 6A] as well as when they were presented across blocks [*r*_(50)_ = −0.297, *p* = 0.03, Figure 6B], further suggesting that lower BDI scores (and presumably higher levels of neurogenesis) are associated with improved pattern separation ability, regardless of whether the similar items are closely or widely spaced in time. We also examined the correlation between BDI scores and our measure of bias toward pattern separation vs. pattern completion described above (number of correct “similar” responses to lure trials minus number of “old” false positives). BDI scores had a significant negative relationship with this measure both within blocks [*r*_(50)_ = −0.284, *p* = 0.04] and across blocks [*r*_(50)_ = −0.408, *p* < 0.01]. In other words, the lower the BDI score, the stronger the tendency is toward pattern separation and the weaker the tendency toward pattern completion; this holds whether the similar items are closely or widely spaced in time. BDI scores did not correlate with the correct identification of novel stimuli or repeated objects within or across blocks. Looking more specifically at performance on the lures that were rated as “less similar” vs. “more similar,” we found that it was performance on the “less similar” items that was most strongly predicted by BDI scores. Within blocks, BDI scores were negatively correlated with the percent of correct classifications of “less similar” lures as “similar” [*r*_(50)_ = −0.334, *p* = 0.02], while being positively correlated with the number of “old” misidentifications of “less similar” lures [*r*_(50)_ = 0.319, *p* = 0.02]. Likewise, across blocks, there was a trend toward a negative correlation between BDI scores and the number of correct selections of “less similar” lures as “similar” [*r*_(50)_ = −0.264, *p* = 0.06], and, conversely, a positive correlation between BDI scores and the proportion of “old” false positive responses to “less similar” lure trials [*r*_(50)_ = 0.284, *p* = 0.04]. Additionally, for “more similar” lures, there was a significant negative correlation between BDI scores and the correct identification of lures appearing across blocks [*r*_(50)_ = −0.299, *p* = 0.03], but no significant correlation for lures occurring within blocks. In sum, those with higher BDI scores made more pattern completion errors (i.e., false positives) at the cost of pattern separation both within and across blocks. This effect was most pronounced when the lure stimuli were considered “less similar” to the target image, due to the fact that more similar lures were rather difficult for both high and low BDI scorers. Importantly, BDI scores did not correlate with performance on paired associates learning, which further argues against the notion that impaired pattern separation in the high BDI group is the result of more generalized deficits in hippocampal-dependent processing.

## Discussion

In Experiment 1, we found that exercise enhanced both fitness levels and performance on a putatively neurogenesis-dependent visual pattern separation task. Specifically, it was those who exhibited the greatest improvement in VO_2peak_, the gold-standard measurement of aerobic fitness, who showed a significant post-exercise enhancement in the ability to distinguish similar lures from previously studied targets. Consistent with these between-group differences, change in fitness taken as a continuous measure significantly correlated with improved pattern separation performance. Mirroring the effects of exercise reported in Experiment 1, we found in Experiment 2 that elevated depression scores negatively predicted visual pattern separation abilities. On the other hand, neither exercise response nor depression scores predicted performance on either the correct classification of repeated items and novel items, or the visuo-spatial PAL task (CANTAB® PAL). Importantly, the CANTAB® PAL task is well-established to be sensitive to hippocampal damage. Thus, both exercise and sub-clinical depression seem to selectively affect pattern separation, rather than having more generalized effects on hippocampal-dependent memory.

As mentioned previously, the non-responder group actually had a somewhat greater level of aerobic fitness as well as pattern separation performance at the onset of our investigation. Accordingly, it may be more accurate to describe the effects of our 6 week exercise intervention as normalizing, rather than enhancing, both fitness and putative neurogenesis-dependent memory function in the responder group. We plan to investigate the relationship between baseline VO_2peak_ and pattern separation performance in future work and expect to find that those with a superior VO_2peak_ score, previously shown to correlate with subjective measures of physical activity (e.g., Herting and Nagel, 2012), will outperform those with lower fitness levels at correctly identifying lures as “similar,” while not differing in their respective ability to detect repetitions or novel objects. This raises the important point that attempts to increase neurogenesis and, therefore, its functional capacity might prove unsuccessful unless there is a deficit to begin with. More generally, the baseline level of neurogenesis must be taken into account. Indeed, in our study it seems that only those with lower levels of fitness, and putatively lower levels of neurogenesis before our exercise training program commenced were the ones who experienced the greatest benefit to putative neurogenesis-dependent cognition following exercise. On the other hand, our attempt to increase neurogenesis in those that had marginally higher levels of fitness and cognitive performance at baseline (non-responders), although no doubt still experiencing some neurogenesis-independent benefits from exercise, was likely redundant with respect to neurogenesis-dependent cognition, as they were already near or beyond the threshold for optimal performance at the onset of our training regime. Future work with the goal of elucidating the relationship between basal neurogenesis and the capacity for change is certainly warranted.

The neural benefits of aerobic exercise, including enhancements in neurogenesis and other physiological and behavioral outcomes, have long been realized (e.g., Cotman et al., 2007; van Praag, 2009). Conversely, it is well-established that high levels of stress contribute to reductions in adult hippocampal neurogenesis (Cameron and Gould, 1994; McEwen, 2001), and to the pathogenesis of major depression (Brown et al., 1999). However, one potential confound in attempting to link depression-related memory deficits to neurogenesis is that stress and depression may lead to broader hippocampal pathology. Conversely, exercise may have widespread effects such as the up-regulation of vasculature and neurotrophins both centrally and peripherally, leading to improved delivery of oxygen and other nutrients to brain tissues etc., and generalized cognitive benefits. Thus, it is important to assess performance on both putatively neurogenesis-dependent memory tasks and other hippocampal-dependent tests of memory, to determine whether lifestyle correlates such as depression and exercise have selective or more generalized cognitive effects. Importantly, meta-analyses suggest that the number of lifetime episodes of major depression predicts the degree of hippocampal atrophy, with first episode sufferers typically showing no signs of hippocampal volume loss (MacQueen et al., 2003; Campbell et al., 2004). We therefore recruited participants in the current study and in our previous study (Becker et al., 2009) who had no prior or current diagnosis of a psychiatric illness and were therefore hypothesized to have reduced neurogenesis as a function of depression scores, in the absence of broader hippocampal pathology. Consistent with this prediction, across the two studies, those with higher depression scores exhibited selective deficits on two different memory tests postulated to be neurogenesis-dependent, in spite of intact performance on a range of other hippocampal- and non-hippocampal-dependent control tasks. Moreover, to our knowledge, the current study is the first to demonstrate opposite effects of chronic exercise and depression levels on performance on a putative neurogenesis-dependent learning task in humans. These results lend evidence to the hypothesis that adult-generated granule cells (or lack thereof) contribute to the cognitive enhancing effects of exercise and the stress-induced impairments in cognition, specifically on a high interference memory task: visual pattern separation.

With lower depression levels, and increased fitness levels, we observed a behavioral shift from pattern completion (erroneous classification of similar items as “old”) to pattern separation (correct classification of similar items as “similar”), presumably due, at least in part, to the increased pool of available newborn cells in the adult DG associated with increased fitness and lower depression scores. This shift can be understood at a neural level, given that young neurons are more plastic and hyperexcitable relative to mature DG neurons (Snyder et al., 2001; Ge et al., 2006; Markwardt et al., 2009), making them more readily available to respond to subtle changes in their input. Furthermore, the young neurons functionally “turnover” by modifying these hyperplastic properties as they grow into maturity (e.g., Becker and Wojtowicz, 2007). In the absence of a pool of young neurons, there would be a shift in bias toward a less excitable and less plastic and static population of DG cells, decreasing the chance of evoking a novel response to a similar, overlapping stimulus in downstream CA3 neurons. Evidence from computational models suggests that when CA3 neurons are only weakly activated by DG inputs, they will respond more strongly and rapidly to their direct EC inputs, shifting their bias toward treating their input as familiar rather than novel, and engaging associative retrieval mechanisms (pattern completion) via their recurrent collateral connections (Nolan et al., 2011). Our findings are consistent with the prediction that exercise shifts the CA3 toward pattern separation, whereas depression levels shift processing toward pattern completion. These findings are also consistent with the well-established effects of ageing on neurogenesis and pattern separation. Age-related declines in neurogenesis have been documented in many species (Heine et al., 2004; Barker et al., 2005; Klempin and Kempermann, 2007; Johnson et al., 2010), and experimental ablation of neurogenesis has been accompanied by a shift from pattern separation to pattern completion in rodents (Clelland et al., 2009). Further, aged humans, who display an age-related decline in neurogenesis similar to that seen in rodents (Knoth et al., 2010), also show deficits on tasks that require pattern separation (Toner et al., 2009; Stark et al., 2010; Yassa et al., 2011; Holden et al., 2012; Stark et al., 2013).

Taken together with the well-established effects of exercise and stress on neurogenesis, and mounting evidence from rodent studies of a critical role for neurogenesis in high interference memory tests (see Introduction), our results are consistent with the hypothesis that neurogenesis is the underlying cause of the exercise-induced enhancement, and the depression-related deficit, in pattern separation that we observed in human participants. As previously alluded to, there was no direct measure of neurogenesis used in our study, therefore, we cannot rule out the possibility that one or more additional variables were affected by exercise and stress, which themselves could have caused or contributed to the observed effects on putative neurogenesis-dependent cognition that are described here. Indeed, both aerobic exercise and stress are known to act on multiple physiological targets (e.g., Brown et al., 1999; McEwen, 2001; Cotman et al., 2007; van Praag, 2009), which include the production of neurotrophins such as brain-derived neurotrophic factor (BDNF) and insulin-like growth factor type-I (IGF-I). Neurotrophins are activity-dependent regulators of brain plasticity. BDNF is increased by aerobic exercise and, while being a well-established positive regulator of neurogenesis in the DG (Sairanen et al., 2005; Scharfman et al., 2005; Henry et al., 2007; Young et al., 2007; Cunha et al., 2010), has also been shown to affect multiple other factors besides the number of adult-born neurons in the subgranular zone. For example, BDNF can influence the release of glutamate and GABA as well as the post-synaptic activation of their receptors which, in turn, can induce calcium influx and influence the cell's basal excitatory postsynaptic potential (Jovanovic et al., 2000; Cunha et al., 2010). BDNF can also activate signal transduction pathways that are important for LTP through binding to its TrkB receptor [reviewed in Cunha et al. (2010)]. All of these changes could contribute to overall brain plasticity and enhanced cognitive performance following exercise. In contrast, a decrease in synaptic plasticity via inhibition of BDNF signaling following chronic stress could contribute to the cognitive deficits observed in those who score high on depression scales. It has been observed that those within a depressed episode show decreased levels of peripheral BDNF in serum (Shimizu et al., 2003). On the other hand, antidepressant pharmaceuticals seem to require TrkB signaling in order to confer behavioral recovery (Li et al., 2008). Peripheral IGF-I is also elevated by aerobic exercise and is required for the running-induced increase in DG neurogenesis (Trejo et al., 2001) and spine density on basal dendrites in the hippocampal CA1 subregion (Glasper et al., 2010). IGF-1 has also been shown to converge on similar pathways as BDNF and has been described as a regulator of exercise-induced BDNF signaling (Ding et al., 2006; Cotman et al., 2007). The increased number of spines on the dendrites of pyramidal cells in the CA1 or IGF-1's effect on BDNF-dependent signaling cascades that lead to increased plasticity in the hippocampus and abroad could be partly responsible for improved hippocampal processing in the absence of changes to hippocampal neurogenesis. Therefore, the putative exercise-induced upregulation of BDNF and/or IGF-1 and depression-associated reduction of BDNF availability could be affecting cellular plasticity in general and cognitive performance in the absence of changes to adult-born neurons in the DG that could, in theory, underlie the changes to learning and memory performance reported here. However, if BDNF and IGF-1 had more pleiotropic effects on cognition, then we would expect to find measureable differences in performance on hippocampal-dependent tasks that are not likely to require neurogenesis, such as CANTAB® PAL. Importantly, we did not observe any significant difference between groups on this particular task. Further, although BDNF and IGF-I can affect factors besides neurogenesis in the subgranular zone, they are nonetheless potent regulators of adult hippocampal neurogenesis. It would thereby be extremely difficult, at least in humans, to parse out or dissociate between the neurogenesis-dependent and -independent contributions of these neurotrophins on cognition. Again, since there was no measure of neurogenesis used here, it is also possible that normalization of pattern separation performance in exercisers and deficits in those with high BDI scores were observed in the absence of changes to neurogenesis. This possibility cannot be completely ruled out without a direct measure of neurogenesis in humans, however, based on the vast number of studies consistently demonstrating an increase in hippocampal neurogenesis following exercise and decrease following chronic stress or stress hormone administration in a variety of non-human species, we can be relatively confident in our assumption that neurogenesis varies between the groups of young adults described here. Breakthroughs in technology and a number of elegant studies would be needed before we can come close to dissociating between the neurogenesis-dependent and -independent effects on cognition in humans and how they might be regulated by, for instance, exercise and stress. In the interim, we have relied on established correlates of neurogenesis from the animal literature. In future work, we plan to elucidate additional biomarkers of neurogenesis in both rodents and humans. Despite the fact that exercise imparts a wide range of benefits on the brain and cognition, this does not mean that DG neurogenesis is not intimately linked to many of these cognitive benefits, and perhaps even its antidepressant effects.

The high intensity interval training manipulation employed here could be more stressful than traditional aerobic exercise training. In general, the intensity and duration of exercise may result in a cost-benefit trade-off. For example, in rodents, a single bout of high intensity exercise sensitizes the organism to the effects of an acute stressor (a radiation challenge), causing elevated markers of oxidative stress, whereas chronic exercise has the opposite effect, inducing protective mechanisms against oxidative stress (de Lisio et al., 2011). Hence, there is a need for more human studies examining the effect of variable durations and intensities of aerobic training in sedentary participants vs., for instance, trained athletes. Specifically, the trade-off in the effects of exercise on positive endpoints such as increased neurotrophins, vs. negative endpoints such as elevated cortisol, inflammatory and oxidative stress markers, as well as on cognitive endpoints, should be investigated. We plan to use salivary cortisol in follow-up experiments to control for stress levels in a non-subjective way.

A limitation of our exercise study is the relatively small sample size, accordingly, the results reported in study 1 should be considered preliminary. While significant effects of exercise on pattern separation were observed, it is possible that with a larger sample exercise might have affected performance on our control tasks as well (correct classification of repetitions and new items, and performance in the PAL task). On the other hand, in a much larger sample of over 50 individuals, we observed a significant relationship between depression scores and pattern separation, but no relationship between depression scores and any of our control tasks. Another caveat is that we included in our non-responder group two participants who did not complete the exercise training. Nonetheless, just looking at the responder group alone, we observed a statistically significant, selective improvement in pattern separation pre- vs. post-exercise with no effect on PAL. We plan to expand on the results of our exercise study in future work by including a non-exercising control group that can be compared to exercisers (regardless of whether they are responders or non-responders), as opposed to performing a median split-based analysis. Though, it should be noted that our median split-based analyses and correlational analyses yielded consistent results.

Another variable that warrants further investigation is the timescale over which exercise or stress may enhance or inhibit neurogenesis and affect memory in humans. The timeline for newly generated granule cells to mature and functionally integrate into the DG-CA3 network in humans is unknown, although estimates from rodents suggest that the process may take 2–4 weeks (Snyder et al., 2009). Evidence from rhesus monkeys suggests that the process may take even longer in primates (Ngwenya et al., 2006). If our exercise program had lasted 12 weeks instead of 6, we may have observed larger or more consistent increases in VO_2peak_ and neurogenesis-dependent memory functions. It has been posited that sustained exercise maximizes the proliferation potential for activity-induced DG neurogenesis, while acute bouts of exercise may only partially restore the neurogenic potential of the subgranular zone (Kempermann, 2010). An important issue is whether a relatively short-term exercise intervention such as the one used here could have comparable effects on neurogenesis and cognition in populations at known risk for depression, such as those with mild depressive symptoms who do not yet meet criteria for a full threshold episode of depression, or even those at high genetic liability. Whether exercise could act via a restoration of neurogenesis to prevent a depressive episode remains to be confirmed, but such an approach has appeal as a health-related disease prevention strategy.

### Conflict of interest statement

The authors declare that the research was conducted in the absence of any commercial or financial relationships that could be construed as a potential conflict of interest.
